# Dynamics of a Distorted Waveform and Elevated Central Venous Pressure (CVP) Resulting From Coiling of a Central Venous Catheter: A Case Report

**DOI:** 10.7759/cureus.83561

**Published:** 2025-05-06

**Authors:** Shashank Paliwal, Jyoti Sharma, Sagar Jolly, Hemanthkumar Tamilchelvan, Navneh Samagh

**Affiliations:** 1 Anaesthesiology, All India Institute of Medical Sciences, Bathinda, Bathinda, IND; 2 Anesthesiology, New York Medical College, Metropolitan Hospital Center, New York, USA

**Keywords:** catheter malposition, central venous cannulation, central venous pressure, coiled catheter, cvp waveform, mechanical complications

## Abstract

Central venous cannulation (CVC) is a routine procedure in critical care and surgical settings. Despite ultrasound guidance, complications like catheter coiling can occur. We report a case of CVC catheter coiling that led to waveform distortion and required corrective measures.

A 23-year-old female patient underwent an emergency laparotomy for perforation peritonitis with underlying pulmonary tuberculosis. During the procedure, a triple-lumen CVC was placed in the left internal jugular vein (IJV) after an unsuccessful right IJV cannulation attempt. Upon placement, the CVP was significantly elevated at 31.2 mmHg with a distorted waveform showing exaggerated 'v wave' and diminished 'x descent'. A point-of-care echocardiogram excluded right heart abnormalities. Postoperatively, a chest X-ray revealed coiling of the CVC in the superior vena cava (SVC), forming a fishhook pattern. The catheter was partially withdrawn by 2 cm, resulting in a reduction in CVP to 12 mmHg and normalization of the waveform. The catheter was subsequently removed and replaced without complications.

Catheter coiling is an uncommon but significant complication of CVC, even when performed under ultrasound guidance. It can lead to inaccurate CVP readings and waveform distortion, which may affect clinical decisions. Early recognition of abnormal waveforms and partial withdrawal of the catheter can correct the coiling and restore accurate hemodynamic monitoring. This case underscores the importance of waveform analysis as a diagnostic tool for detecting catheter malposition and mechanical complications.

## Introduction

Central venous cannulation (CVC) is a routinely performed procedure in operating rooms and intensive care units (ICU), typically via internal jugular, subclavian, or femoral veins. It enables reliable venous access for administering medications, fluids, hemodynamic monitoring, blood sampling, dialysis, and emergency interventions for resuscitation. Despite advancements in ultrasound (US) guidance, CVC continues to present technical challenges and complications, including catheter malposition, hematoma, pneumothorax, accidental arterial puncture, and in rare cases, coiling or looping of the catheter [1]. We report an uncommon case of a coiled left internal jugular vein (IJV) catheter, resulting in elevated central venous pressure (CVP) and a distorted waveform.

## Case presentation

A 23-year-old female patient (weight, 45 kg; height, 160 cm) presented for an emergency laparotomy due to perforation peritonitis and underlying pulmonary tuberculosis. On arrival, she was in shock, with a heart rate of 183 bpm, blood pressure of 70/40 mmHg, and an international normalized ratio (INR) of 1.6. Following fluid resuscitation, she underwent general endotracheal anesthesia with rapid sequence induction with intravenous etomidate and succinylcholine.

**Figure 1:**
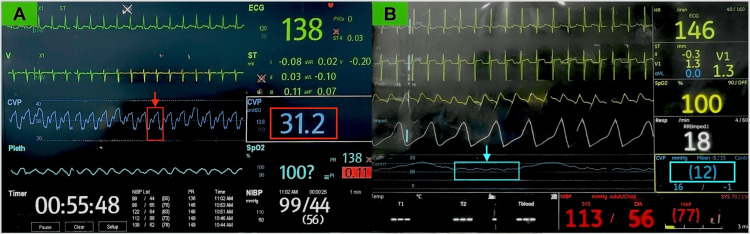
(A) Intraoperative elevated CVP with distorted waveform showing exaggerated 'v' wave and diminished 'x' descent (red arrow) due to coiled CVC. (B) Postoperative normalization of CVP and waveform (blue arrow) after partial catheter withdrawal. CVP: central venous pressure.

**Figure 2:**
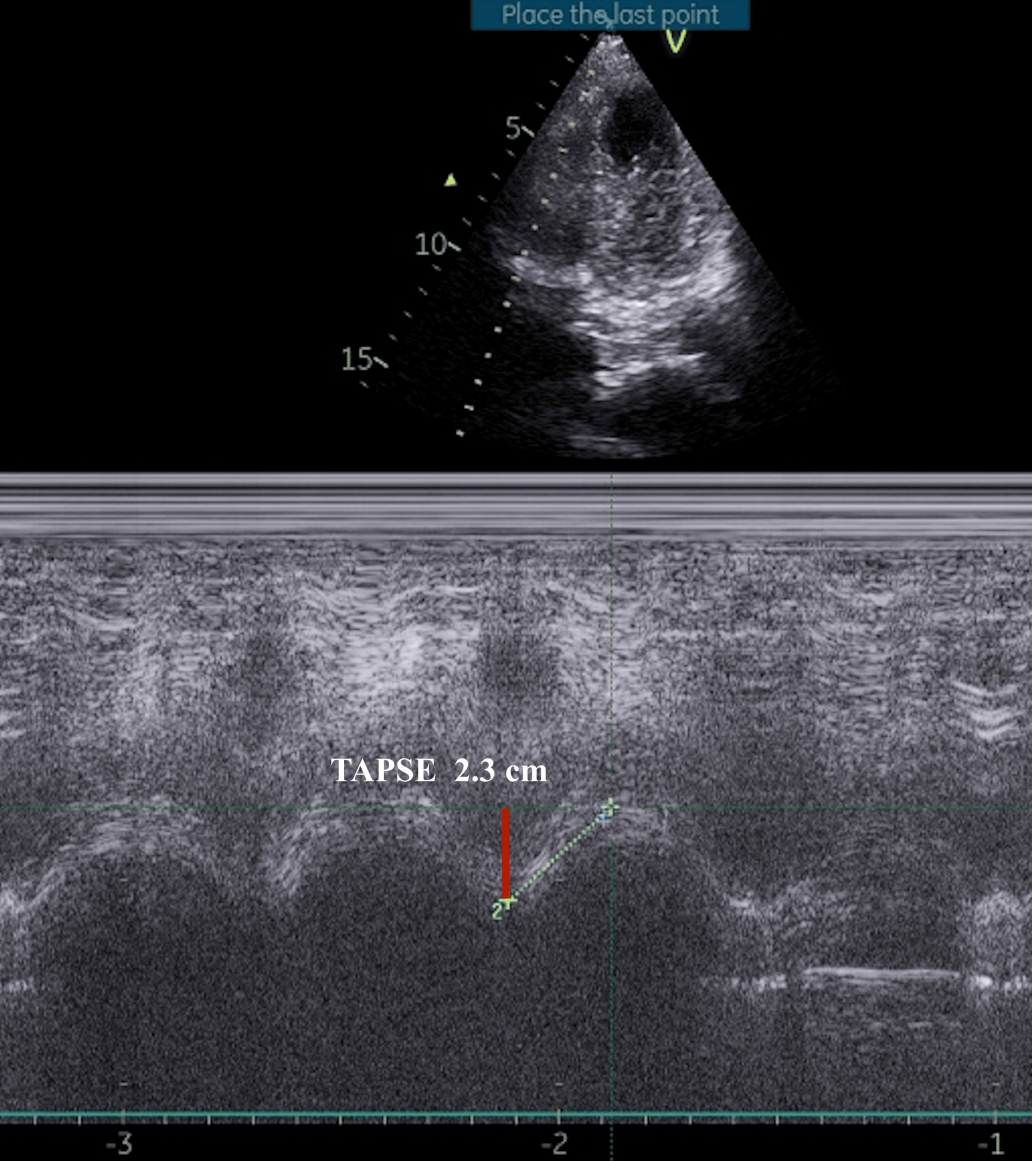
Point-of-care ultrasound derived M-mode echocardiography showing preserved right ventricular systolic function with TAPSE (red line) measurement of 2.3 cm. TAPSE: Tricuspid annular plane systolic excursion.

A US-guided right internal jugular CVC placement was attempted initially but was unsuccessful due to the inability to negotiate the guidewire on the side. Subsequently, a triple-lumen CVC (16-18-18G, 7 Fr, 20 cm, Certofix®, B. Braun, Melsungen, Germany) was successfully placed in the left IJV using the Seldinger technique, advancing to the 15 cm mark. During guidewire withdrawal, it got momentarily stuck, and a snap occurred with minimal force, though the wire was fully retrieved intact, and all catheter ports aspirated blood appropriately without resistance. The measured CVP was significantly elevated at 31.2 mmHg with a distorted waveform (Figure 1A), initially presumed due to transducer or line malfunction, but equipment checks confirmed normal function. A point-of-care echocardiogram ruled out right heart abnormalities and the patient proceeded for surgery (Figure 2).

**Figure 3:**
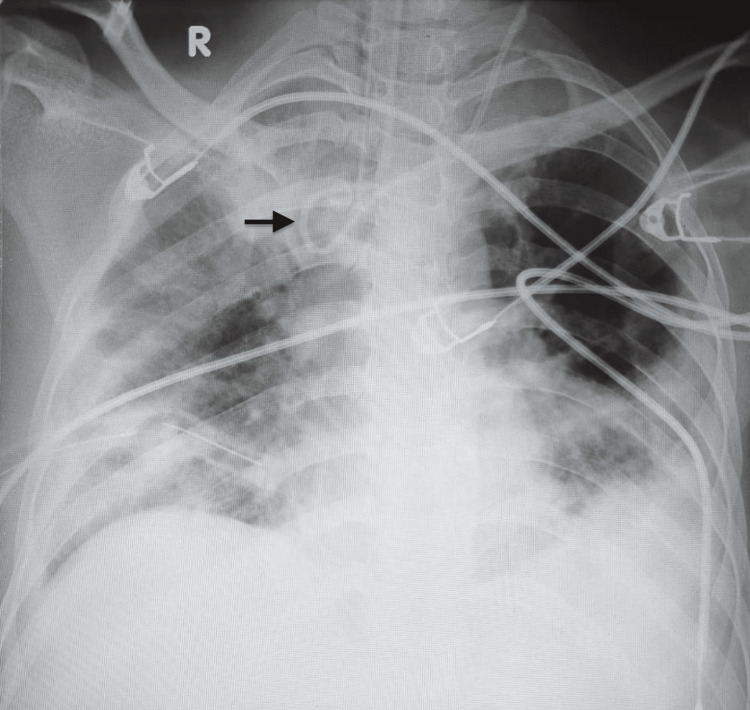
Postoperative chest X-ray showing coiled left internal jugular venous catheter tip (black arrow) forming a fishhook pattern.

Intraoperatively, the patient received 2 liters of crystalloids, two units of packed red blood cells (PRBC), two units of fresh frozen plasma (FFP), and inotropic support. Postoperatively, she was transferred intubated to the surgical ICU, maintaining inotropic support via the CVC. A chest X-ray revealed the catheter was coiled in a fishhook pattern within the superior vena cava (Figure 3). The CVC was withdrawn by 2 cm, resulting in a decrease in CVP to 12 mmHg with a normalized waveform (Figure 1B). The catheter was subsequently removed without resistance or damage, and a new left IJV catheter was successfully placed under US guidance with appropriate blood aspiration from all ports without resistance.

## Discussion

In hospitals where real-time US guidance is standard for CVC, mechanical complications occur in approximately 7% of cases, with 0.4% being significant complications such as bleeding, arrhythmias, arterial puncture, catheter malposition, and pneumothorax [1]. Factors such as BMI<20, multiple punctures, and subclavian vein catheterization increase the risk of these complications [1]. A 2024 meta-analysis of 214,325 central venous catheterizations found a 3% overall serious complication rate, including arterial cannulation, pneumothorax, bloodstream infection, deep vein thrombosis, and placement failure. Subclavian access had the highest estimated risk of pneumothorax and placement failure, while internal jugular access had the highest rate of bloodstream infection. However, overlapping credible intervals limited definitive comparisons between access sites [2].

CVC coiling and looping have been reported with both internal jugular and subclavian vein access [3-5]. Malposition occurs more frequently with left IJV (4.12%) than right IJV (1.1%) and is more common with soft silicone than semi-rigid catheters [6]. A radiographic review of 378 central venous catheterizations found a 5.3% incidence of malposition and loop formation, with looping alone in 2.9%, most often via the external jugular (30%) and internal jugular (5.7%) veins [7]. Similarly, a meta-analysis of 2,602 CVC placements reported a malposition rate of 6.8%, highlighting the persistence of this complication despite ultrasound guidance [8].

Excessive guidewire or catheter insertion length, forceful guidewire withdrawal, and directing the J-tip of the guidewire cephalad during subclavian vein access can cause coiling or misplacement of CVC [9,10]. Guidewire insertion in adults generally should not exceed 18 cm [9]. In our case of left IJV cannulation, a 15 cm insertion was adequate, although resistance and a snapping sensation were encountered during withdrawal. The guidewire was intact, but the potential locking of the J-tip with the catheter tip may have contributed to looping despite proper initial positioning.

Catheter coiling in the SVC or IJV can distort CVP waveforms by impeding blood flow. Normal CVP waveforms display a, c, and v waves along with x and y descents. If the catheter is coiled, the pressure signal may not be transmitted effectively, as seen with our case, which can lead to reduced or absent 'a' wave, eliminating or blunting the 'x' descent, exaggerated 'v' wave and a normal 'y' descent depending on the obstruction caused by coiling as shown in Figure 1A. Withdrawal of the catheter by 2 cm normalized the waveform, as shown in Figure 1B. Similarly, Goyal and Sahu have also reported dampening of the CVP waveform with falsely elevated CVP due to a coiled CVC in the left subclavian vein [11]. In line with these findings, Chennakeshavallu et al. described a malpositioned catheter tip abutting the SVC wall, producing a waveform mimicking ECG activity, which normalized after withdrawing the catheter by 1 cm [12].

CVP waveform analysis is a rapid and reliable method for early detection of catheter malposition, especially during IJV cannulation, with a demonstrated sensitivity of 97.5% and specificity of 100%, making it a valuable tool for real-time clinical decision-making [13]. In our case, we could aspirate blood from all three ports of the coiled catheter without any resistance. However, the CVP waveform showed a normal 'a' wave followed by an exaggerated 'v' wave and an absent 'x' descent. Normally, the 'x' descent reflects a drop in right atrial pressure during atrial relaxation and downward movement of the tricuspid valve. When a catheter is coiled, pressure transmission from the right atrium to the tip can be delayed or distorted, leading to a diminished or absent 'x' descent.

The coiled catheter can create resistance that prevents the pressure from properly reflecting the atrial relaxation. The 'v' wave corresponds to the venous filling of the right atrium when the tricuspid valve is closed. In the presence of coiling, the catheter can act like a partial obstruction, increasing right atrial pressure as blood accumulates during systole (venous return phase), which appears as a more prominent 'v' wave on the CVP waveform. These distorted pressure dynamics can prevent the expected pressure drop during atrial relaxation while exaggerating the pressure during atrial filling. The 'y' descent represents passive emptying, in contrast to the forceful atrial contraction of the 'a' wave. If the catheter is coiled in a way that does not fully occlude the venous pathway, passive blood transfer from the atrium to the ventricle may still occur, resulting in a normal or near-normal 'y' descent. It can be seen that coiling often affects phases where blood flow depends more on active contraction or changes in intrathoracic pressure, such as during atrial contraction or systole. However, during diastole (the relaxation phase), the right atrium passively empties into the ventricle, and a partially coiled catheter may not impede this process enough to alter the 'y' descent on the waveform.

While US guidance has made venous access safer by improving visualization during puncture, it does not eliminate the risk of catheter tip malposition. Intra-atrial ECG offers a useful method by tracing P wave changes with increased amplitude or bifid shape as the catheter enters the right atrium, returning to normal shape once positioned at the cavoatrial junction [14]. Techniques such as contrast-enhanced ultrasound, the saline flush test with rapid atrial swirl sign (RASS), defined as turbulence in the right atrium within 2 seconds after flushing the distal port of the CVC, intra-atrial ECG, and height-based formulas including Peres’ method (height/10 + 4 cm for the left IJV, with adjustments for other sites), Czepizak’s method (height/10 - 1 cm), Lum’s method (height/10 - 1.3 cm), Lee’s method (height/10 - 1.5 cm), and Kim’s fixed-depth method (15 cm) have all contributed to faster and more accurate confirmation of catheter tip positioning near the cavoatrial junction [15-18]. More recently, external-landmark methods using anatomical surface markers, such as the distance from the puncture site to the upper edge of the right third costosternal junction, have also been introduced for bedside guidance [19]. However, while these methods confirm where the tip ends, they provide little insight into how the catheter traveled through the vessel. As a result, mechanical issues like looping, kinking, or coiling can easily go unnoticed without direct imaging, which can be best visualized through imaging techniques like chest radiography or transesophageal echocardiography (TEE). Routine chest X-rays or TEE are not always feasible in the operating room. In such situations, CVP waveform interpretation can reveal the coiling of the catheter, as seen in our case with elevated CVP and a distorted waveform, which can be corrected by repositioning or re-inserting a new CVC under US guidance.

## Conclusions

CVC coiling is uncommon but can occur despite US guidance. This can lead to falsely elevated CVP values with distorted waveform, seen as an exaggerated 'v' wave and diminished 'x' descent. Early recognition of abnormal waveforms and careful partial catheter withdrawal can restore accurate CVP measurements. Therefore, CVP waveform analysis can help recognize mechanical complications such as coiling of the catheter.
